# Promoting sustainable research partnerships: a mixed-method evaluation of a United Kingdom–Africa capacity strengthening award scheme

**DOI:** 10.1186/s12961-015-0071-2

**Published:** 2015-12-23

**Authors:** Laura Dean, Janet Njelesani, Helen Smith, Imelda Bates

**Affiliations:** Liverpool School of Tropical Medicine (LSTM), Liverpool, UK; New York Institute of Technology (NYIT), New York, USA; Centre for Maternal and Newborn Health, Liverpool School of Tropical Medicine, Liverpool, UK

**Keywords:** Capacity strengthening, International collaborations, Low- and middle-income countries, Mixed methods

## Abstract

**Background:**

Research partnerships between high-income countries (HICs) and low- or middle-income countries (LMICs) are a leading model in research capacity strengthening activities. Although numerous frameworks and guiding principles for effective research partnerships exist, few include the perspective of the LMIC partner. This paper draws out lessons for establishing and maintaining successful research collaborations, based on partnership dynamics, from the perspectives of both HIC and LMIC stakeholders through the evaluation of a research capacity strengthening partnership award scheme.

**Methods:**

A mixed-method retrospective evaluation approach was used. Initially, a cross-sectional survey was administered to all award holders, which focused on partnership outputs and continuation. Fifty individuals were purposively selected to participate in interviews or focus group discussions from 12 different institutions in HICs and LMICs; the sample included the research investigators, research assistants, laboratory scientists and post-doctoral students. The evaluation collected data on critical elements of research partnership dynamics such as research outputs, nature of the partnership, future plans and research capacity. Quantitative data were analysed descriptively and qualitative data were analysed using an iterative framework approach.

**Results:**

The majority of United Kingdom and African award holders stated they would like to pursue future collaborations together. Key aspects within partnerships that appeared to influence this were; the perceived benefits of the partnership at the individual and institutional level such as publication of papers or collaborative grants; ability to influence ‘research culture’ and instigate critical thinking among mid-career researchers; previous working relationships, for example supervisor-student relationships; and equity within partnerships linked to partnership formation and experience of United Kingdom partners within LMICs. Factors which may hinder development of long term partnerships were also identified such as financial control or differing expectations of partners.

**Conclusions:**

This paper provides evidence of what encourages international research partnerships for capacity strengthening to continue past award tenure, from the perspective of researchers in high and LMICs. Although every partnership is unique and individual experiences subjective, this paper provides extension and support of key principles and mechanisms that can contribute to successful research partnerships between researchers.

## Background

Research capacity strengthening is defined as a ‘process of individual and institutional development which leads to higher levels of skills and greater ability to perform useful research’ [1]. Research partnerships between high-income (HIC) and low- and middle-income countries (LMICs) have become a leading model in the implementation of research capacity strengthening activities [2, 3]. As the partnership approach becomes favourable to funders, such as the United Kingdom’s Department for International Development (e.g. African Capacity Building Initiative) and the European Union (e.g. European and Developing Countries Clinical Trials Partnership), it is crucial to understand how to nurture successful partnerships to ensure achievement of intended outcomes and value for money. The partnership approach focuses on mutual capacity enhancement and two way flow of knowledge between research institutions and their staff in HICs and LMICs [4, 5]. The approach emphasises mutual trust and shared decision making as opposed to older models of capacity strengthening where knowledge transfer was unidirectional [6]. Perceived benefits of the partnership approach to researchers in high-income settings are often cited as deeper contextual understandings of working in LMICs, as well as possibilities for reverse innovation whereby processes learnt in LMICs can be adapted and implemented in HICs [7]. Benefits to researchers in LMIC settings include the opportunity to use research outcomes to develop evidence-based policy and programming, as well as contribute to a growing population of research scientists [8]. Partnerships can lead to a more equitable environment for research, where it is more likely that individual knowledge and skills can be translated into sustainable institutional capacity to generate and disseminate knowledge [8, 9]. Given the potential benefits to all partners, it is important to explore what factors contribute to successful research partnerships and are likely to promote such ongoing collaborations.

Recent research has led to a proliferation of frameworks and principles outlining the characteristics of effective research partnerships [10]; however, few explore partnerships from the perspective of the LMIC partner. Even when the views of researchers from LMICs are examined within such frameworks, this is often limited to leading partners and neglects the viewpoints of all actors within the partnership, including students, grant makers, research councils and administrative departments [11]. Such frameworks are often not informed by interdisciplinary dialogue which would generate lesson learning about how research partnerships may operate in different research environments [11]. Until partnership frameworks include the views of all stakeholders and interdisciplinary dialogue exists, they are unlikely to offer much guidance to researchers, funders or policymakers in planning for fair and equitable research partnerships that promote mutual capacity strengthening [12].

In 2008, an award scheme was launched aimed at supporting universities and institutes in Africa to develop sustainable research partnerships and research training capacity. The objectives of the scheme were (1) to strengthen research capacity of institutions in a specific West African country and a specific East African country; (2) to enhance research training including PhD programmes in these countries; and (3) to improve international collaborative capabilities of institutions in these countries. The awards provided 3 years of funding to scientists to develop a collaborative research project between the United Kingdom and research institutions in either the West or East African country identified. The scheme covered all areas of the life and physical sciences, with a focus on national research priorities in selected countries including basic human health research, agriculture, water and sanitation, biodiversity, and energy. The scheme has been delivered in two phases of funding, with 18 awards granted in phase one (2008–2012) and five in phase two (2012–2016). In 2013, the Capacity Research Unit at the Liverpool School of Tropical Medicine conducted a retrospective evaluation of the scheme to determine if the award scheme had been successful in meeting its three objectives and to guide planning for future schemes.

This paper draws out lessons for research partnerships from the evaluation findings. With a specific focus on partnership dynamics, it highlights the factors that have influenced the ability of researchers in African and United Kingdom institutions to establish and maintain research collaborations, with the aim of increasing knowledge about how to successfully promote such partnerships. Our evaluation included researchers, staff and post-graduate students involved in the award in United Kingdom and African institutions, to identify what they perceived would contribute to the long-term success or failure of the established collaborations.

## Methods

The evaluation was retrospective [13], and used qualitative and quantitative data collection methods. We used semi-structured interviews and focus group discussions (FGDs) to gather the perceptions and views of the people and institutions involved in the award initiative.

The online cross-sectional survey collected data on three main areas: (1) research outputs at partnership level; (2) partnerships and collaborations; and (3) sustainable collaborations. Sustainability referred to the partnership continuing beyond the current award funding period. These areas were designed to allow the evaluation team to explore how individual partnership dynamics influenced the achievement of the awards third objective: to improve international collaborative capabilities of institutions (with a specific focus on researchers in the institution) in the selected countries.

### Sampling

To identify participants for interview, a purposive sample was taken from a list of award principal investigators (PIs) and co-principal investigators (Co-PIs). PIs were always based in United Kingdom institutions and Co-PIs were always based in African institutions. PIs and Co-PIs are together referred to as award holders. The sample aimed to achieve maximum variation in geographic location of institute, sex and seniority of award holder, and award research topic as the award scheme engaged with various scientific areas from a variety of disciplines. Within the African institutions visited, to generate multiple perspectives of how partnerships operated, opportunistic and snowball sampling was used to identify other award stakeholders at Co-PIs’ institutions for face-to-face interviews. These stakeholders included university deans/principles, research staff, laboratory technicians and administrative staff. Opportunistic sampling was also used to identify students to take part in FGDs within LMIC institutions. We included students (PhD or Masters) or research staff working on the award, whilst aiming for maximum variation relating to sex, award research area and role in the award partnership.

### Data collection

All data collection was completed between March and September 2013. Qualitative data were collected by JN, who visited research partners in the West African country, East African country, and the United Kingdom to conduct interviews and FGDs at the participants’ institution. Where interviews could not be conducted face to face, they were conducted by telephone or computer via Internet voice calls. Semi-structured interviews and FGDs were conducted using a topic guide which covered several areas: research outputs, nature of the partnership (e.g. origins, what works, what does not work), future plans, and research capacity, identified as critical for effective research partnerships. We also explored collaborative outputs that the award had mobilised. Interviews were recorded with the permission of the interviewee. An observer made notes on the dialogue during FGDs. Both interviews and FGDs were conducted in English.

For the survey, we used the following inclusion criteria for participants: award holders who had access to a computer and had provided an email address to the awarding body, and were able to complete the survey in English. All award holders who met the inclusion criteria were a contacted via email and asked to complete the survey electronically. To maximise response rate, participants were recruited using the Modified Dillman approach [14]. This involved the awarding body sending a pre-email to potential participants followed by emails being sent every 2 weeks from the Capacity Research Unit to encourage participation in the survey, this continued for 6 weeks until the survey closed [14].

The survey took approximately 15 minutes to complete and was administered through Bristol Online Survey. Surveys were initially piloted on award management staff. The final survey was sent out via email and completed electronically within an 8-week timeframe.

### Analysis

In-depth interviews and FGDs were transcribed verbatim and analysed thematically using a Framework approach [15]. To ensure rigour within analysis, coded data were peer checked amongst the research team [15]. Cross sectional survey data were analysed descriptively using the Bristol Online Survey and MS Excel programs. Given that all of the data were rated on nominal or ordinal scales, results are presented as frequencies and percentages.

### Ethical statement

Ethical approval was obtained from the Liverpool School of Tropical Medicine ethics committee (ref: 13.21). The award funder, participants and institutions have been anonymised in this paper. All participants were provided with written and verbal information about the evaluation, which included the evaluation process, the purpose and nature of the evaluation, their right to withdraw at any time, guidelines for withdrawal, how confidentiality will be maintained, the risks and benefits of participating, and contact information of the investigators. Survey data were collected anonymously using the Bristol Online Survey and stored in password protected files. In relation to qualitative data, transcripts and audio recordings were held in password protected files and all data are reported anonymously.

## Results

### Survey findings

The survey link was emailed to a total of 43 award holders, of which 53% (n = 23) responded. Of these, 61% (n = 14) were the African partner (Table 1). Survey data are presented under two key areas – continuing collaborations and benefits of the partnership which includes research outputs.

**Table 1:** Participant characteristics: cross sectional survey

	Awardee (EAC/WAC), n = 14 (%)	Awardee (UK), n = 9 (%)
Sex		
Male	12 (85.7)	5 (55.5)
Female	2 (14.3)	4 (44.4)
Age, years		
≤45	4 (28.6)	2 (22.2)
>45	10 (71.4)	7 (77.7)
Place of work		
Research institute	4 (28.6)	1 (11.1)
University/education institute	10 (71.4)	8 (88.8)
Main work role		
Non-research	9 (64.3)	4 (44.4)
Research	5 (35.7)	5 (55.5)

EAC, East African Country; UK, United Kingdom; WAC, West African Country

### Continuing collaborations

Survey data provided information about the likelihood of continuing collaboration as assessed by the participants as a result of this award. The majority of United Kingdom award holders (77.8%, n = 7) and African award holders (78.6%, n = 11) stated they would like to pursue future collaborations together. As evidence of this commitment, survey data showed that the majority of award holders had already investigated their next collaborative funding opportunity (Tables 2 and 3). It highlighted that international funding bodies were the most appealing, particularly to the African partner, with 78.6% (n = 11) of these respondents stating that they are likely to pursue this as a future funding source.

**Table 2:** Collaborative research outputs of award holders since successful application

	Award holder, n = 23
Publications jointly authored^a^	
0	2 (8.6)
<50%	0 (0)
≥50%	4 (17.3)
100%	5 (21.7)
No publications	6 (26.1)
Grants awarded in collaboration with partner institute^b^	
0	6 (26.1)
<50%	0 (0)
≥50%	0 (0)
100%	3 (13.0)
No grants	5 (21.7)
Conference presentations in collaboration with partner institute^c^	
0	5 (21.7)
<50%	0 (0)
≥50%	0 (0)
100%	9 (39.1)
No conference presentations	4 (17.3)

^a^No response, n = 6; ^b^No response, n = 9; ^c^No response, n = 5

**Table 3:** Potential for future collaborations and the perceived benefits from present collaboration

	Awardee (EAC/WAC), n = 14 (%)	Award holder (UK), n = 9 (%)
Future collaborations		
Yes	11 (78.6)	7 (77.8)
No	1 (7.1)	0 (0)
Undecided	2 (14.3)	2 (22.2)
Types of collaboration		
Jointly develop teaching and training	7 (50.0)	3 (33.3)
Staff academic or teaching exchanges	5 (35.7)	4 (44.4)
Joint PhD supervision	10 (71.4)	6 (66.6)
Level of collaboration		
Institutional	3 (21.4)	2 (22.2)
Departmental	2 (14.3)	0 (0)
Individual	1 (7.1)	4 (44.4)
Combination	8 (57.1)	3 (33.3)
Future grant collaborations		
Yes	11 (78.6)	7 (77.8)
No	0 (0)	0 (0)
Undecided	3 (21.4)	2 (22.2)
Future funding sources		
National funding bodies	1 (7.1)	5 (55.5)
International funding bodies	11 (78.6)	5 (55.5)
National government institution or university funds	2 (14.3)	3 (33.3)
Collaboration benefits		
Additional general financial support from UK institution	5 (35.7)	4 (44.4)
Additional general financial support from African institution	1 (7.1)	1 (11.1)
PhD bursaries from UK institution	1 (7.1)	3 (33.3)
PhD bursaries from African institution	0 (0)	2 (22.2)
Course fee waivers UK institution	3 (21.4)	2 (22.2)
Course fee waivers African institution	1 (7.1)	0 (0)
Sharing of lab space, research space, equipment	8 (57.1)	5 (55.5)

EAC, East African Country; UK, United Kingdom; WAC, West African Country

### Benefits of the partnership

Survey data quantified the personal benefits of participating in research partnerships, specifically the opportunity to produce research outputs. These are outlined in Table 3 and include publications, conference presentations and being awarded future grants. The majority of conference presentations and paper publications resulting from the award were achieved in conjunction with the collaborating partner. Conversely, the majority of additional grants awarded had been achieved independently of the collaborative partner (Table 2). Specifically, only three of the additional nine grants gained by awardees since receiving the award had been received collaboratively, three were obtained independently by the African partner and the remaining three were obtained independently by the United Kingdom partner. Participants also identified other financial income as a result of the partnership as a key benefit; 44% (n = 4) of United Kingdom award holders and 35.7% (n = 5) of African award holders mentioned receiving funds outside of the grant from a United Kingdom institution as a benefit of the award. As well as institutional benefits in the form of finance, survey data also revealed that United Kingdom award holders (55%, n = 5) and African award holders (57.1%, n = 8) considered sharing laboratory space and research equipment as a collaborative benefit.

### Qualitative findings

In total, 42 people participated in semi-structured interviews or FGDs from 12 different institutions (Table 4), including PIs, Co-PIs, research assistants, students and laboratory scientists. We identified four main themes through the analysis using the Framework approach. These relate to factors that could influence the nature, the outputs, or the sustainability of the partnerships.

**Table 4:** Participant characteristics: site visits

	Interviewee (UK), n = 5 (%)	Interviewee (EAC), n = 13 (%)	Interviewee (WAC), n = 24 (%)
Sex			
Male	2 (40.0)	12 (92.3)	19 (79.0)
Female	2 (40.0)	1 (7.7)	5 (20.8)
Age, years			
Under 25	0 (0)	0 (0)	1 (4.2)
25–35	0 (0)	1 (8.3)	9 (37.5)
36–45	2 (40.0)	2 (15.4)	5 (20.8)
46–55	2 (40.0)	7 (53.8)	3 (12.5)
56–65	1 (20.0)	1 (8.3)	2 (8.3)
>65	0 (0.0)	1 (8.3)	0 (0)
Unknown	0 (0.0)	1 (8.3)	4 (16.7)
Role			
Principal investigator	5 (100.0)	5 (38.5)	5 (20.8)
Head of department/college	0 (0)	4 (30.1)	5 (20.8)
Research Staff^a^	0 (0)	1 (7.7)	5 (20.8)
PhD student	0 (0)	1 (7.7)	4 (16.7)
Masters student	0 (0)	2 (15.4)	3 (12.5)
Unknown	0 (0)	0 (0)	2 (8.3)

^a^Research staff includes senior researchers, research assistants and laboratory technicians. EAC, East African Country; UK, United Kingdom; WAC, West African Country

### Collaborative benefits and career progression

During interviews and FGDs, the majority of West and East African researchers identified benefits of the partnership to both themselves and their students, such as the learning and teaching they received from the United Kingdom partners. In FGDs, some students also identified this as beneficial:

“*With the partnership I think it is so far so good because a professor from the UK has been involved in several training sessions on this campus each time that he came…. So, so far the partnership has been good and beneficial to us as students….I think as early-stage researchers this opportunity has been one of the best opportunities that we have probably ever had or I have ever had.*” (West African Masters Student, Female, Under 25).

“*For capacity building we have a number of trainings going on and our students and junior staff will benefit from the training that we are going to offer…it’s really just knowledge transfer I think that’s going to be very successful.*” (East African PI, Male, 36–45).

The ability to produce papers and present at conferences as a result of the award was also cited as a key individual benefit and a key contribution to individual career progression:

“*…we have to be able to write papers and this is one of the benefits. This can be used for our promotion.*” (West African PI, Female, 56–65).

At the institutional level, institute leaders and award PI’s felt that working with a United Kingdom partner and the prestige of the award benefitted their institutions’ reputation. They also felt that the prestige of the award allowed them to engage more senior staff within the institution. They perceived that this had a positive influence on capacity strengthening activities as these staff members were often key institutional decision makers:

“*…. it gives them the institutional recognition that somebody has this award and this is prestigious so that is important…. so it creates a culture of people who can apply and get grants.*” (West African PI, Male, Age 36–45).

Participants from the United Kingdom and the African institutions both perceived that the benefits of the award scheme accrued primarily for the African partner. Some of the United Kingdom PIs we spoke to felt they had also benefitted from participating in the partnership, especially the experience of working in LMICs, but the majority tended to identify contributions they had made to the African partner and institution:

“[I was] *able to contribute to a larger proposal as I had partnerships in place and I could use this* [LMIC] *experience to receive other awards.*” (United Kingdom PI, Female, 46–55).

### Research culture

In some cases, the critical elements needed to strengthen ‘research culture’ at institutions (i.e. protected time for research, types of learning pedagogies, training in research methods, journal clubs) were lacking, and participants described the impact of this on achieving effective partnerships and high quality outputs as it was “*often hard to find people who are even committed to research post Masters*” (United Kingdom PI, Female, 36–45). However, several United Kingdom PI’s recognised that the partnership had enabled them to begin to support junior researchers towards independence, but acknowledged this takes time and requires institutions to invest more in strengthening research culture:

“*When you go to* [XXXX] *meetings it focuses on scientific excellence….You cannot change the whole philosophy of an institution can just plant that seed and hopefully some of those will continue to be associated with you and your research. It has to be an organic thing from within. The Director is very enlightened and has those ideas in his head. Try to reach maximum number of students. How do they go on to become independent researchers? Few become independent researchers, but I’m sure we’ve had an impact. But we need to expand that, through this or other African funding. It is really worth continuing as the real impact is in the continuation to change research cultures and philosophy.*” (United Kingdom PI, Female, 46–55).

Impact on ‘research culture’ was described as being most noticeable when mid-level post-doctoral researchers were involved in partnerships, since they had time to invest in the research process as well as the energy and motivation to influence change at the institutional level. The PI’s and co-PI’s we spoke to believed there were increasing numbers of ‘home-grown’ post-doctoral researchers who needed support and encouragement. It was perceived that engaging them in award schemes such as this had a positive impact on research culture through exposure to international collaboration and funding opportunities, as well as opportunity to develop project expertise and confidence to produce scientific outputs:

“*I know what it is like when research is not paramount, it can stall growth and it contributes to brain drain, post-doctoral culture is growing so it is important we encourage dialogue with them.*” (United Kingdom PI, Female, 46–55).

“*I’ve nearly always found that the higher level people are the pains to work with but actually the middle level people are the ones who have the umph to get past the base level but haven’t got to the higher levels are usually the ones who can be the most dynamic and put the most effort into it.*” (United Kingdom PI, Female, 46–55).

### Partnership functionality and sustainability

When asked what had made partnerships effective (i.e. function as a partnership) some participants identified communication as a key factor. Key markers of effectiveness were described to participants as joint idea development, joint decision making and joint research outputs. All the postgraduate students we interviewed felt that regular interaction and communication with the United Kingdom partner assisted them in development of learning. Consequently, when communication was ineffective, it was perceived as having detrimental impact on the collaboration:

“*The scientific work has been okay, communication has not been good,* [and] *this is not the best in terms of collaborating with other partners. There has been a lack of response as in you send emails and you don’t get any response and that sort of thing and then they come back and say they’re sorry but I just think that they are all excuses … It all has to do with communication. I would say* [X] *is a very good scientist, but the communication could have been better. I think that is the key thing in terms of collaboration and communication. The way we share data, it all comes back down to communication.*” (West African PI, Male, 46–55).

Some PI’s and co-PI’s and other researchers involved in the award felt that more important than communication to achieving effective authentic partnerships, was the way in which the partnership was formed. Most of these participants believed that pre-existing relationships between partners, such as a former PhD supervisor-student relationship, often resulted in more successful project outcomes:

“…*well for the PI in the UK we have known for two years and each year they will be here doing work or training programs with us. …. I think the partnership has been really, really fantastic. So I think that that is good.*” (West African Researcher, Male, 25–35).

“*The UK PI, Prof* [X]*, trained me, I was his Ph.D. student so I worked with him for five years no sorry for four years…it was smooth and swift, because this is my work I sat down and invented it kind of, and then I was able to convince him to come on board using techniques that he had taught me so it made the transition swift and it has been easy. We talk as scientists at that level where when no one has thought about the other because the thing is it’s just a suggestion and there is no ‘no no no’. We don’t have that.*” (West African Researcher, Male, 36–45).

Award holders interviewed reported that they would like to pursue future collaborations together and they had already investigated their next collaborative funding opportunity:

“*There is a proposal about bio-char* [being developed] *so we are in touch about that.*” (East African PI, Female, 56–65).

### Equitable partnerships

The PIs and researchers we interviewed stressed the importance of equity between partners and the influence this had on research outputs, partnership benefits and the potential for future collaborations. Some reported that when partners entered the project with differing assumptions about, for example, the research content and focus, the administration of funds, or procurement process the effect on the partnership was debilitating. Sometimes, a lack of knowledge about the research context in LMICs was a barrier to effective working within a partnership:

“…*so what* [X] *was saying you see,* [X] *has her style of working of course it is different than what we are used to doing. We said we don’t take credit cards here the system for procurement at the university; we have to follow the procedures which are allowed but* [X] *say we don’t know and we should learn how to use credit cards…So this is the problems we have and of course there is not much progress in this context in terms of achievements.*” (East African PI, Male, 46–55).

“*…there wasn’t any money for overheads, we weren’t allowed to charge any overheads whatsoever, so I had to create a negotiation with the institution and eventually I did pay 5% which I thought was the least but I had to support it with money from elsewhere because the* [X] *itself did not allow us to charge any overheads whatsoever for administering, which you need for such a grants, you need someone to help to keep all the records. So that is the bits that I would say could be improved.*” (West African PI, Male, 46–55).

The administrative staff we spoke to who were engaged with the award highlighted administrative aspects of award management, such as where funds were held as underlying factors in creating inequitable partnerships. They explained that despite the majority of the money (50–70%) being spent in the African countries, the African institutions had limited or no autonomy in relation to when and how funds were spent. Several PI’s and Co-PI’s and administrative award staff identified that allowing the African partner to have more financial control would create more equitable partnerships. They also felt that this would reduce delays associated with international bank transfers, and complex administrative processes between the United Kingdom and African partners. Some researchers described having to use existing grants to finance initial activities in the award scheme, because of delays in transfer of funds from the United Kingdom:

“*…the only problem we have had is that we still don’t have the actual money hit our account yet but because we have other grants I’ve been able to support the work…I would suggest that 20% of the money is made directly…*[to] *the African researchers* [they] *can have some start-up money… and then the UK partners can handle the bulk of the money because you have seen what I’m going through because I’ve if I didn’t have another grant we wouldn’t be able to do anything.*” (West African Researcher, Male, 36–45).

Participants said they tended to encounter problems in achieving equitable partnerships when the partnership was new, and no prior collaboration had taken place. Several participants felt that inequity was more likely to occur when United Kingdom based partners had no or limited experience of carrying out research in LMICs:

‘*I do think it helps if you have worked in LMICs even if it is not in* [East African country] *directly. Just that understanding that things don’t work the same as they do here is really beneficial, I am not saying that it should be restricted to those sorts of people because that’s not fair because people can adjust and they can learn….I don’t really know how to prepare people for that though, other than perhaps talking to somebody who has done it, that’s probably the only useful thing.*” (United Kingdom PI, Female, 46–55)

## Discussion

Overall, the majority of evaluation participants reported that the partnership they were involved with as a result of this award scheme had the potential to continue. Key aspects within partnership dynamics that appeared to influence this were the perceived benefits of the partnership to individuals and institutions, ability to influence ‘research culture’ and support junior and mid-career researchers to develop skills and confidence, previous working relationships, and equity within partnerships linked to partnership formation and researcher experience working in LMICs. Factors which may hinder long-term partnerships developing were also identified, such as financial control or partners having different expectations when embarking on new partnerships.

This evaluation was retrospective and it was not possible to explore temporal elements of partnership development; this may have provided greater insight into how aspects of effective partnerships develop over time. Because it was retrospective, we had no information about the capacity of the award holders and their institutions at baseline (i.e. before the award began). As a result, we could only explore partnerships in relation to their immediate outcomes and outputs and it is too soon to make any inference about the long-term impact or sustainability of the award partnerships. Furthermore, a lot of the potential these partnerships show for continuation is self-reported and therefore findings must be interpreted cautiously. The survey response rate was low (53%), which may add some response bias to the findings; however, triangulation of findings through qualitative data helps to minimise this bias. More men than women participated in the survey and the qualitative component, reflecting the composition of award holders, with more awards held by men than women. We tried to include female participants where possible, but we were unable to recruit comparable numbers of women and men. The fact that the evaluation team comprised researchers from a high-income setting may give a cultural bias to the interpretation of evaluation findings. However, the evaluation team has a wealth of experience working in LMICs so it is anticipated that their contextual understandings would allow for reflexivity to minimise this bias.

Qualitative findings reflected that United Kingdom partners were unable to identify many benefits of the partnership to them, whilst African partners identified several benefits. When asked in the survey about partnership benefits and research outputs, however, both United Kingdom partners and African partners were able to identify outputs that appear beneficial to a research career regardless of geographic location. Afsana et al. [16] suggest that “*effective partnerships will only succeed if all parties are truly engaged in a way that is just and beneficial*”. Implying, therefore, that if HIC partners are unable to identify the benefit research partnerships have for them the ‘effectiveness’ and potential sustainability of the partnership is challenged. Imbalance in perceived benefits between research partners in HICs and LMICs also raises issues of power dynamics within the partnership, which could limit the authenticity of the partnership [6]. In order to encourage authentic partnerships, these findings support and extend Syed et al.’s [7] argument suggesting that, whilst more can be done to identify benefits to researchers in high-income settings, work also needs to be done to change the thinking of those researchers so that they are able to identify these benefits when they exist. If, however, there are limited benefits to the HIC partner, it may improve partnership dynamics to be honest about this at partnership conception so that partners’ assumptions are realistic and equity within relationships is ensured. Finally, it supports Bradley’s [11] argument that measuring success of partnerships via output measures alone such as ‘co-authorship of peer reviewed publication’ is unlikely to provide true representation of partnership dynamics. Until more research is conducted into benefits to partners from HICs, funders could make assessment of this by encouraging them to make reference to perceived personal and institutional benefits as part of application and reporting processes. Focused research that is able to engage with research partnerships at the beginning of award tenure would allow for more critical exploration of the motivations of participants and institutions, especially those based in the United Kingdom, for engaging in award schemes such as the one evaluated here. This would likely provide strong contributions to understanding perceived benefits to all partners and thus contribute to achieving equitable partnerships.

The findings reported herein support some of the principles of good partnerships outlined in guidelines for sustainable partnership formation [5, 11, 17]. Findings support and extend theoretical frameworks that shape the overarching partnership principles by providing practical examples which evidence their necessity as highlighted in Table 5. Practical examples are derived from reflecting on participants’ tacit knowledge having been involved in research partnerships over a number of years. Our findings also allow for pragmatic recommendations to be made about measures that can be put in place to promote previously identified ‘good’ partnership principles (Table 5). To achieve authenticity and equity it may not always be possible for new partnerships to be based on existing links and previous experience of working in LMICs; however, mentoring by those with more partnership or LMIC experience could increase the potential for equity and authenticity. In addition, funding agencies could make more rigorous assessment of the level of engagement both parties have had in research design to ensure priorities of both researchers in HICs and LMICs and their institutions are fulfilled [18]. Throughout the course of award tenure, funding agencies could also monitor frequency, type and effectiveness of communications as a means to assess partnership relationships.

**Table 5:** Matching existing partnership principles with this study’s pragmatic examples to promote effective relationships

Principles of a good partnership *Adapted from* [5, 17]	Practical examples from this study	Recommendations to promote ‘effective’ partnerships
Set the Agenda Together	Communication perceived as crucial to promoting authenticity in partnerships	Encourage frequent communication through various methods including Skype/telephone and face to face meetings
A broad based consultation should precede any programme	Previous working relationships advantageous factor in effective working relationships	Funders can provide networking opportunities as pre-cursors to partnership awards to build working relationships and contextual understandings
Interact with stakeholders	Important to have the same assumptions when entering partnership to ensure equity or effects can be debilitating	Funders can make assessment of engagement of all partners/stakeholders in study design and implementation plans
	Where experience of LMIC context was limited, inequity in partnerships was more likely to occur based on lack of contextual understandings	Establishment of mentorship schemes for researchers in HICs with limited experience in LMICs to improve contextual understandings
Clarify responsibilities	African institutes would like more financial control	Simultaneous strengthening of financial systems in LMIC institutions accompanied by change in award financial regulations to give LMIC partners more financial control
The northern partner should be prepared to relinquish control and to accept considerable autonomy on the part of the Southern partner	Decision-making between Southern and Northern partners should be equitable with complementary roles; this will reduce or eliminate power imbalances	
Promote mutual learning	Benefits mainly identified by the UK PI as to what they had provided to the African PI with the exception of learning about LMIC context	Funders and award partners should be explicit about the benefits to themselves of North–South research partnerships
	African researchers perceived teaching received by UK partners as beneficial to their learning	Work with Northern partners to encourage them to identify potential learning opportunities for themselves within the partnership
Enhance capacities	UK and African award holders perceived sharing of laboratory space and research equipment as a collaborative benefit	Incorporate strengthening of institutional infrastructures so that partnership benefits can be sustained
Strengthening capacities to produce socially relevant research should be a specific aim of the partnership	Additional grants received independently by the African partner	Whilst collaboration is critical to successful partnerships, encourage partners to establish grant diversity and resilience
Share data and networks	Majority of conference presentations and paper publications resultant from the award were collaborative	Promote collaborative dissemination of research findings through different mechanisms
Disseminate results		
Pool profits and merits		
Apply results		
Secure outcomes		

HIC, High-income countries; LMICs, Low- and middle-income countries; PI, Principal investigator; UK, United Kingdom

Baud [19] suggested greater exploration of modalities of successful partnerships, specifically their process and structures. These findings highlight that the way in which funds are managed cannot only impede research implementation but also cause inequities that impact upon the potential for partnership success. Changing award structures to give more financial autonomy and control to LMIC institutions would likely improve the equity between partners and in turn is likely to contribute to potential successful and sustainable partnerships [18]. In order to change funding structures, however, funders need to be confident that organisations have robust financial and auditing systems. This would suggest that it is not as simple as changing award implementation structures but also the need to engage with LMIC partners to strengthen administrative systems, particularly those related to financial management [20]. The way finances are controlled is important to ensure equitable relationships for capacity strengthening. Strengthening financial systems is a key component of institutional capacity, allowing institutions to better manage research income. Funders and researchers could pay more attention to this aspect, for example, by facilitating bespoke training or sharing lessons on financial management.

This study has contributed toward a deeper understanding of how to generate effective research partnerships as well as providing some practical examples. However, to further understand partnerships for research capacity strengthening, longitudinal studies that observe partnership dynamics as they develop and identify critical factors that contribute to their success or failure would provide more insight. Detailed understanding of the benefits to HIC partners in being involved in schemes such as this would also assist in identifying motivations for their engagement. Finally, studies that compare award administrative structures including how finances are managed between LMIC and HIC partners may improve implementation of award schemes and increase both research and capacity strengthening outcomes.

## Conclusions

This evaluation provides evidence of what facilitates international partnerships for research capacity strengthening to continue past award tenure. The evaluation includes the perspectives of both HIC and LMIC researchers and a variety of stakeholders whilst incorporating opinions from different scientific disciplines. Although the evaluation was of a specific award scheme that prioritised research in the life and physical sciences, it is likely that researchers in other disciplines will recognise aspects of the partnerships we highlight in the results. Whilst it is appreciated that every partnership is unique and individual experiences subjective, our findings support key principles and mechanisms that can contribute to successful research partnerships.

## Abbreviations

Co-PI: Co- Principal Investigator
FGD: Focus group discussion
HIC: High-income countries
LMICs: Low- and middle-income countries
PI: Principal Investigator
